# Results from a randomized controlled trial testing *StressProffen*; an application‐based stress‐management intervention for cancer survivors

**DOI:** 10.1002/cam4.3000

**Published:** 2020-04-03

**Authors:** Elin Børøsund, Shawna L. Ehlers, Cecilie Varsi, Matthew M. Clark, Michael A. Andrykowski, Milada Cvancarova, Lise Solberg Nes

**Affiliations:** ^1^ Department of Digital Health Research Division of Medicine Oslo University Hospital Oslo Norway; ^2^ Department of Psychiatry and Psychology College of Medicine and Science Mayo Clinic Rochester Minnesota; ^3^ Department of Behavioral Science College of Medicine University of Kentucky Lexington Kentucky; ^4^ Faculty of Health Sciences Oslo Metropolitan University Oslo Norway; ^5^ Institute of Clinical Medicine Faculty of Medicine University of Oslo Oslo Norway

**Keywords:** cancer, cancer survivor, cognitive behavioral, digital, distress, health‐care delivery, psycho‐oncology, stress‐management

## Abstract

**Background:**

In‐person cognitive‐behavioral stress‐management interventions are consistently associated with reduced cancer distress. However, face‐to‐face delivery is an access barrier for many patients, and there is a need to develop remote‐delivered interventions. The current study evaluated the preliminary efficacy of an application (app)‐based cancer stress‐management intervention, *StressProffen*, in a randomized controlled trial.

**Methods:**

Cancer survivors, maximum 1‐year posttreatment (N = 172), were randomized to *StressProffen* (n = 84) or a usual care control group (n = 88). Participants received a blended delivery care model: (a) one face‐to‐face introduction session, (b) 10 app‐based cognitive‐behavioral stress‐management modules, and (c) follow‐up phone calls at weeks 2‐3 and 6‐7. Outcome measures included *stress* (Perceived Stress Scale), *anxiety* and *depression* (Hospital Anxiety Depression Scale), and *health‐related quality of life* (HRQoL; Short‐Form Health Surveys [SF‐36]) at 3‐months post‐intervention, analyzed with change scores as dependent variables in linear regression models.

**Results:**

Participants were primarily women (82%), aged 20‐78 years (mean 52, SD 11.2), with mixed cancer types (majority breast cancer; 48%). Analysis of 149 participants completing questionnaires at baseline and 3 months revealed significant intervention effects: decreased *stress* (mean difference [MD] −2.8; 95% confidence interval [CI], [−5.2 to −0.4]; *P *= .022) and improved *HRQoL* (Role Physical MD = 17.7, [CI 3.7‐31.3], *P *= .013; Social Functioning MD = 8.5, [CI 0.7‐16.2], *P *= .034; Role Emotional MD = 19.5, [CI 3.7‐35.2], *P *= .016; Mental Health MD = 6.7, [CI 1.7‐11.6], *P *= .009). No significant changes were observed for *anxiety* or *depression.*

**Conclusions:**

Digital‐based cancer stress‐management interventions, such as *StressProffen*, have the potential to provide easily accessible, effective psychosocial support for cancer survivors.

## Graphical abstract

In a randomized controlled trial, cancer survivors with access to an app‐based cognitive‐behavioral stress‐management intervention (*StressProffen)* compared with a control group reported a significant reduction in perceived stress level and a significant increase in health‐related quality of life at 3 months. Interventions such as *StressProffen*, with easy access and use, have the potential to provide low cost, highly accessible psychosocial support for a large group of cancer survivors.

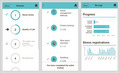

## 1 INTRODUCTION

A cancer diagnosis and the ensuing treatment are often associated with substantial physical and psychosocial challenges, including fatigue, discomfort, pain, stress, distress, anxiety, and depression.^1^, ^2^, ^3^, ^4^, ^5^ Such symptom burden is associated with reduced ability to self‐regulate emotions, cognitions, and behavior as well,^6^ and coping can be challenging.^4^, ^7^

More than 30 years of research demonstrate the efficacy of cognitive‐behavioral cancer distress‐ and stress‐management interventions to reduce stress, anxiety, depression, and improve quality of life (QoL) and social support.^1^, ^3^, ^8^, ^9^, ^10^, ^11^, ^12^, ^13^, ^14^ Positive long‐term effects of such psychological interventions for cancer survivors have also been observed.^15^ Unfortunately, many access barriers exist for such interventions, including (a) interventions typically being limited to large, urban medical centers, (b) limited insurance coverage, and (c) cancer survivors not feeling physically or emotionally well enough to attend in‐person sessions.^12^ These barriers, together with high rates of unmet needs with regard to rehabilitation and psychosocial support,^16^ identify a critical need to expand health‐care delivery options for cancer survivors.

Remote health‐care delivery options for cancer distress‐ and stress‐management are in their infancy. Results from peer‐reviewed published randomized controlled trials (RCTs) examining telephone interventions show mixed results,^17^ and even though telephone‐based interventions may improve accessibility, barriers related to the cost of therapist time, insurance coverage, and the need for set appointment times still remain. eHealth interventions (eg, web‐based, application [app]‐based) may reduce such barriers. However, eHealth interventions for cancer survivors are still at an early stage, published results from RCTs testing psychosocial eHealth interventions are scarce, and recent findings remain mixed and overall inconclusive.^18^, ^19^, ^20^, ^21^

Examining the current status of digital interventions delivering psychosocial support to cancer survivors, scientific reviews have highlighted a need to focus on design and adaptation of evidence‐based interventions, while involving patients and health‐care provider stakeholders in the development process.^18^, ^22^ Other reviews have indicated that digital interventions aiming for individualization and improving coping skills appear most likely to have positive impact for cancer survivors,^23^ and that professionally guided interventions may have better effect than self‐guided interventions.^24^ Furthermore, attrition has emerged as a challenging issue for digital interventions, and attention to intervention adherence at an early stage has emerged as an important factor of success for digital interventions.^20^, ^25^

Based on existing research and the potential of psychosocial eHealth interventions to reach a high number of cancer survivors, the current research team developed *StressProffen* ^TM©^, an app‐based cognitive‐behavioral stress‐management intervention program for cancer survivors.^26^, ^27^ The current study aimed to examine preliminary results from an RCT testing the *StressProffen* intervention program. It was hypothesized that participants receiving the *StressProffen* intervention, compared to participants in a usual care control group, would at 3 months post‐intervention initiation experience *decreased perceived stress* (primary outcome), *improved health‐related quality of life *(*HRQoL*), and *decreased anxiety and depression* (all secondary outcomes).

## 2 MATERIALS AND METHODS

### 2.1 Design

A two‐armed RCT‐assigned participating cancer survivors to; (a) an app‐based stress‐management intervention (*StressProffen*) or (b) a usual care control group.

### 2.2 Participants and recruitment

Participants were patients diagnosed with any type or stage of cancer, recruited at a major medical center in Northern Europe or through social media, between June 2017 and July 2019. Eligibility criteria: (a) currently or recently in cancer treatment (maximum 1 year since hospital treatment completion); (b) ≥18 years of age; (c) able to speak, read, and understand Norwegian; (d) access to smartphone or tablet; and (e) able to attend one face‐to‐face introduction session.

### 2.3 Study procedure

The study was approved by institutional research review bodies: Regional Committee for Medical and Health Research Ethics (approval number will be inserted) and the Hospital Privacy Protection Committee (approval number will be inserted). The study was registered in ClinicalTrials (Clinicaltrials.gov: NCT02939612).

Patients were informed about the study either orally or through flyers by staff at out‐patient clinics and radiotherapy units. If patients were interested, the staff forwarded their contact information to study personnel, who then contacted the patients and provided additional information about the study. Some of the interested patients contacted study personnel on their own, either by phone or through a study web page. In addition, the medical center published Facebook and Instagram postings about the study. These social media postings were reposted by several cancer‐specific patient organizations, and patients interested in participating then contacted the study personnel either by phone or through the study web page. All participants provided written informed consent, then completed baseline questionnaires before randomization. Computerized randomization allocated study arms 1:1 (block size 10) stratified by gender and diagnosis (breast cancer vs all other diagnoses based on experience from the pilot study,^27^ where the largest group (40%) of cancer diagnoses was women with breast cancer).

Outcome measures (for both groups) were completed online and submitted electronically through a secure server using an encrypted connection. The respondents could submit their data via computer, smart phone, or tablet. Server log data (eg, duration and number of times participants signed into their app) were automatically collected electronically through a secure server, also using an encrypted connection. Participation included completing outcome measures at baseline and at 3 months. In addition, the intervention group received; (a) a face‐to‐face introduction session; (b) 10 app‐based thematic modules; and (c) follow‐up phone calls at 2‐3 and 6‐7 weeks post introduction session. Program completers were defined as participants completing at least 70% (7 of 10) of the modules.^28^

### 2.4 Description of the *StressProffen* intervention

The design and development of *StressProffen*, reported elsewhere,^26^ were based on evidence‐based intervention components tested within gold‐standard cognitive‐behavioral distress‐ and stress‐management interventions,^1^, ^9^, ^11^, ^12^, ^14^ utilizing user‐centered design methods with close collaboration between scientists, cancer survivors (“users”), psychosocial‐oncology health‐care providers, system developers, and eHealth experts.^26^ To properly evaluate the effectiveness of complex interventions, the Medical Research Council recommends initial intervention testing and refinement to ensure intervention feasibility.^29^ A single‐arm pilot examining system use, usefulness, ease of use, and preliminary effects of the *StressProffen* intervention program has therefore previously been conducted^27^ to inform optimization and preparation for a full‐scale RCT. Participating cancer survivors in the previously conducted pilot test described *StressProffen* as providing a new, appreciated, and easily accessible stress‐management tool, while simple pre‐post intervention analyses showed significant decreases in stress, anxiety, and self‐regulatory fatigue, as well as improved HRQoL.^27^

The *StressProffen* intervention included individually tailored content as well as professional contact (ie, a face‐to‐face introduction visit and two follow‐up phone calls). The introductory session was conducted as a one‐time face‐to‐face in‐person structured, manuscripted, individual or group session (ie, practical decision by the research team, depending on how many participants were available to attend at the time of each session) led by health‐care study personnel trained by a licensed clinical health psychologist. The session introduced participants to stress‐management concepts, provided help downloading the *StressProffen* app, and provided professionally guided practice in how to use the app. Participants were encouraged to complete all 10 modules and also to practice content for at least 30 minutes per day in order to benefit as much as possible from the program. They were also informed that the first four modules were sequential, while the order of modules 5‐9 could be individually chosen. Participants were also informed that in order to encourage content practice, each thematic module would have to be open for 3 days before the next module could be opened. Participants could at any point chose between reading and listening, and preferred exercises could be marked with a “My favorite” button for easy retrieval and practice. The follow‐up phone calls at 2‐3 and 6‐7 weeks were administered by health‐care study personnel trained by a licensed clinical health psychologist. They followed a structured manuscript including open questions about the participants’ impression of the app so far, and whether the participants had had any problems using the program. The first and/or last authors were part of the initial introductory sessions and follow‐up phone calls to ensure fidelity to the intervention protocol. In addition, project team, including the licensed clinical health psychologist (ie, last author), met weekly to further ensure fidelity to the intervention protocol for the introductory sessions and follow‐up phone calls. For more details about *StressProffen* please see Børøsund and colleagues 2018, 2019.^26^, ^27^

**FIGURE 1:**
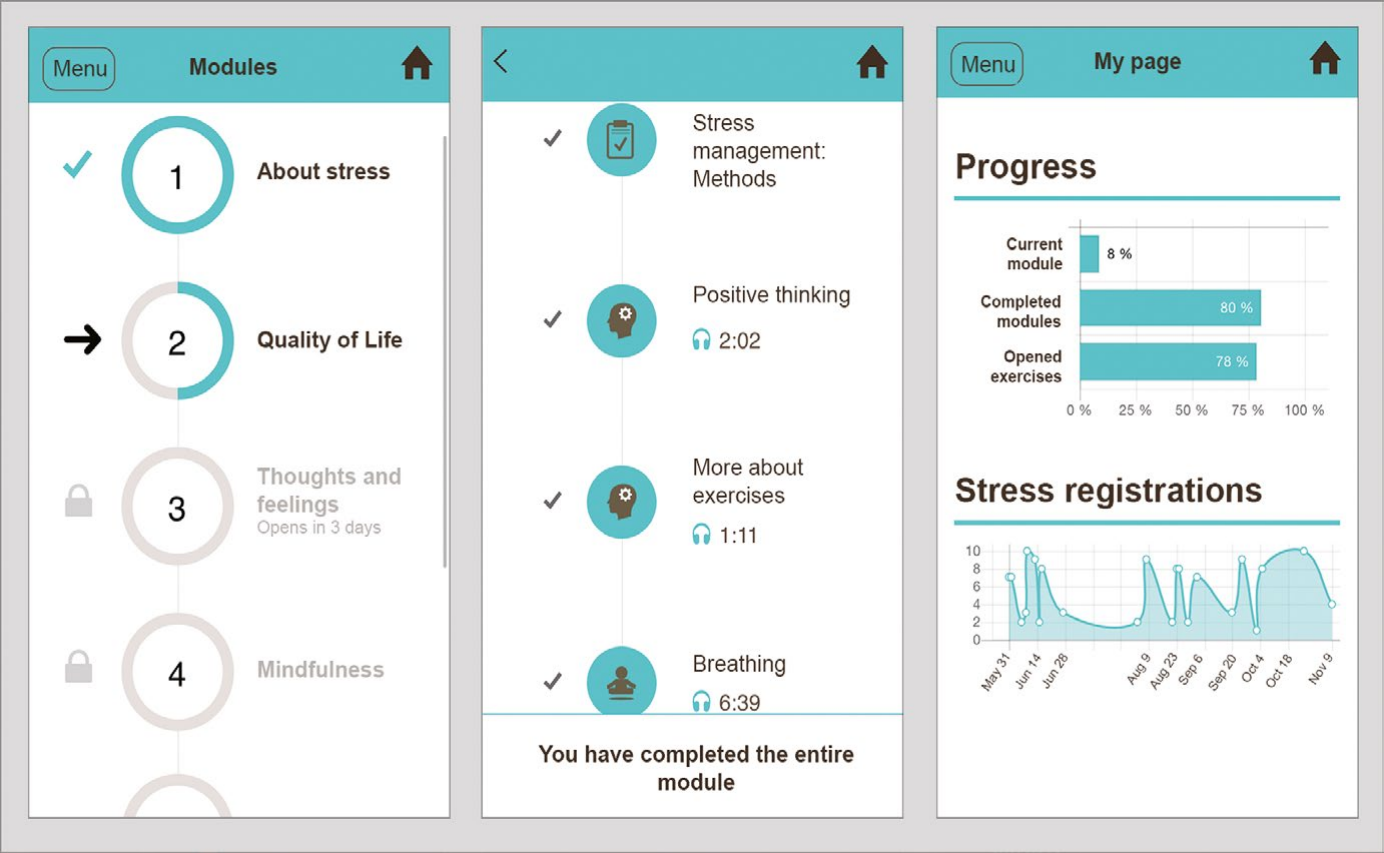
Examples of *StressProffen* screenshots

The 10 modules in *StressProffen* are also described elsewhere^26^ and included the following themes (see Figure 1 for screenshots): (a) What is stress; (b) stress, QoL and planning; (c) thoughts, feelings and self‐care; (d) mindfulness, rational thought‐replacement and guided imagery; (e) stress and coping; (f) Social support, humor and meditation; (g) anger management and conflict style awareness; (h) assertiveness and communication; (i) health behaviors and setting goals; and (j) review and summary. Participants could choose between reading and listening to the program at any time. The program was optimized for smartphones as well as tablets. Participants could contact the study staff on weekdays for questions through a project phone number.

### 2.5 Data collection and outcome measures

Demographic data, disease, and treatment information were collected at baseline through a study specific self‐report questionnaire (see Table 1). Comorbidity was measured with The Self‐Administered Comorbidity Questionnaire (SCQ‐19),^30^ a self‐report questionnaire assessing 16 common and three optional medical conditions. The total SCQ‐19 score can range from 0 to 57, and a higher score indicates a more severe comorbidity profile.

**TABLE 1:** Baseline sociodemographic‐ and disease‐related measures (N = 172)

Characteristics	All participants N = 172	Intervention group n = 84	Control group n = 88	*P* value
Age, mean (SD)	52 (11.3)	51.7 (10.5)	52.3 (12.0)	.725
Gender, n (%)				
Female	141 (82)	69 (82)	72 (82)	.956
Male	31 (18)	15 (18)	16 (18)	
Marital status, n (%)				
Married/cohabitating	120 (70)	56 (67)	64 (73)	.387
Single/divorced	52 (30)	28 (33)	24 (27)	
Education, n (%)				
Elementary/high school	33 (19)	17 (20)	16 (18)	.943
University/college ≤4 years	60 (35)	29 (35)	31 (35)	
University/college >4 years	79 (46)	38 (45)	41 (47)	
Household annual income, (EUR^a^), n (%)				
<40 000	18 (11)	9 (11)	9 (10)	.629
40 000‐60 000	32 (19)	15 (18)	17 (19)	
60 000‐80 000	16 (9)	5 (6)	11 (13)	
80 000‐1 00 000	31 (18)	17 (20)	14 (16)	
>100 000	75 (44)	(45)	37 (42)	
Employment status, n (%)^b^				
Full‐time/part‐time work	36 (21)	18 (21)	18 (21)	.334
Sick leave/disability benefits	120 (70)	61 (73)	59 (67)	
Retired/other	16 (9)	5 (6)	11 (13)	
Treatment^c^, n (%)				
Operation	126 (73)	66 (79)	60 (68)	.124
Chemotherapy	102 (59)	46 (55)	56 (64)	.236
Hormone therapy	44 (26)	21 (25)	23 (26)	.864
Radiation	74 (43)	34 (41)	40 (46)	.519
Immune therapy	18 (11)	8 (10)	10 (11)	.694
Other	24 (14)	10 (12)	14 (16)	.449
Diagnosis, n (%)^c^				
Breast cancer	83 (48)	39 (46)	44 (50)	.639
Brain cancer	13 (8)	9 (11)	4 (5)	.126
Prostate cancer	10 (6)	6 (7)	4 (5)	.467
Lymphoma	8 (5)	4 (5)	4 (5)	.946
Colon cancer	8 (5)	4 (5)	4 (5)	.946
Other	50 (29)	22 (26)	28 (32)	.417
Metastases, n (%)	23 (13)	12 (14)	11 (13)	.731
Months since diagnosis, median (range)	8 (0.25‐240)	7.0 (0.25‐120)	8.5 (0.25‐240)	.183
Comorbidity, median (range)	3 (0‐20)	3.0 (0‐20)	3.0 (0‐17)	.467

^a^ EUR = 1 EURO is approximately 1.1 USD; approximately 10 Norwegian kroner (fall 2019).

^b^ Percentages not 100 due to rounding.

^c^ Participant could receive several treatments.

#### 2.5.1 Psychosocial outcome measures

##### Primary outcome

*Perceived stress *was measured by the Perceived Stress Scale (PSS‐14), a 14‐item scale measuring feelings and thoughts over the last month.^31^ Items were rated on a 5‐point Likert scale, ranging from “Never” (0) to “Very often” (4). Total PSS‐14 score can range from 0 to 56. Higher scores indicated higher perceived stress. The PSS‐14 has no cutoff scores as it is not a diagnostic instrument, but scores are referred to as low, moderate, and high.

##### Secondary outcomes

*Anxiety and depression* were measured with the Hospital Anxiety and Depression Scale (HADS)^32^; a 14‐item measure of anxiety and depression, validated as a unidimensional measure of distress. Items were rated on a 4‐point scale (0‐3), with a total score range from 0 to 42. The HADS is divided into two subscales: Anxiety (HADS‐A; 7 items) and depression (HADS‐D; 7 items). Scores below 8 are considered nonclinical, scores 8‐11 as suggestive of the presence of anxiety/depression, and scores above 11 as definite presence of anxiety/depression. There are, however, some indications that these cutoff numbers could be too high for cancer patients, resulting in underrecognition of distress.^33^

*Health‐related quality of life *was measured with the noncommercial SF‐36‐Item Short‐Form Health Survey (RAND‐36 version),^34^, ^35^ a 36‐item measure of physical‐, role‐, emotional‐, cognitive‐, and social function, as well as physical health, general, and global health/HRQoL. Scores can range between 0 and 100 for all subscales, with the lower the score the more disability (0 = maximum disability, 100 = no disability). A mean score of 50 has been articulated as a normative value for all scales. While a mean of 50 has been estimated as normative in the general US population, the normative for a Norwegian sample is somewhat higher.^36^

#### 2.5.2 System use, usefulness, and ease of use

System use log data, including details of use and program progress, were extracted from user logs stored on a secure research server. Post‐intervention, participants rated intervention acceptability and feasibility using three Likert ratings scaled from 1—“totally agree”—to 5—“totally disagree”: (a) the program was easy to use, (b) the exercises were easy to understand, and (c) the program was useful.

### 2.6 Statistical analyses

Baseline characteristics, usefulness/ease of use, and user patterns summarized with mean and standard deviation (SD) for normally distributed variables, and medians and range for non‐normally distributed variables are presented. Categorical data are presented as counts and percentages. Change scores from baseline to 3 months were calculated for perceived stress, anxiety, depression, and HRQoL and used as dependent variables in linear regression models. As no statistically significant differences were observed between the intervention and the usual care control groups related to demographic‐ and disease‐related factors at baseline, no possible confounders were included in the intention‐to‐treat analysis. The models were initially adjusted for age and gender, but removed from the final model as they were not statistically significantly correlated with the outcome. Models were fitted both with change score as the dependent, and with the outcome (eg, perceived stress at 3 months) as the dependent adjusted for baseline as a covariate. For the second model, there was a need for more statistical power given the adjustment for an additional covariate, meaning somewhat less precision in the estimates. Model fit was tested by means of visual inspection of histograms of residuals and was deemed as a very good fit for the models with change scores as the dependent. *P*‐values <.05 were considered statistically significant. Statistical analyses were completed using the Statistical Package for the Social Sciences (release 24; SPSS Inc., Chicago, IL, USA).

## 3 RESULTS

### 3.1 Sample description

**FIGURE 2:**
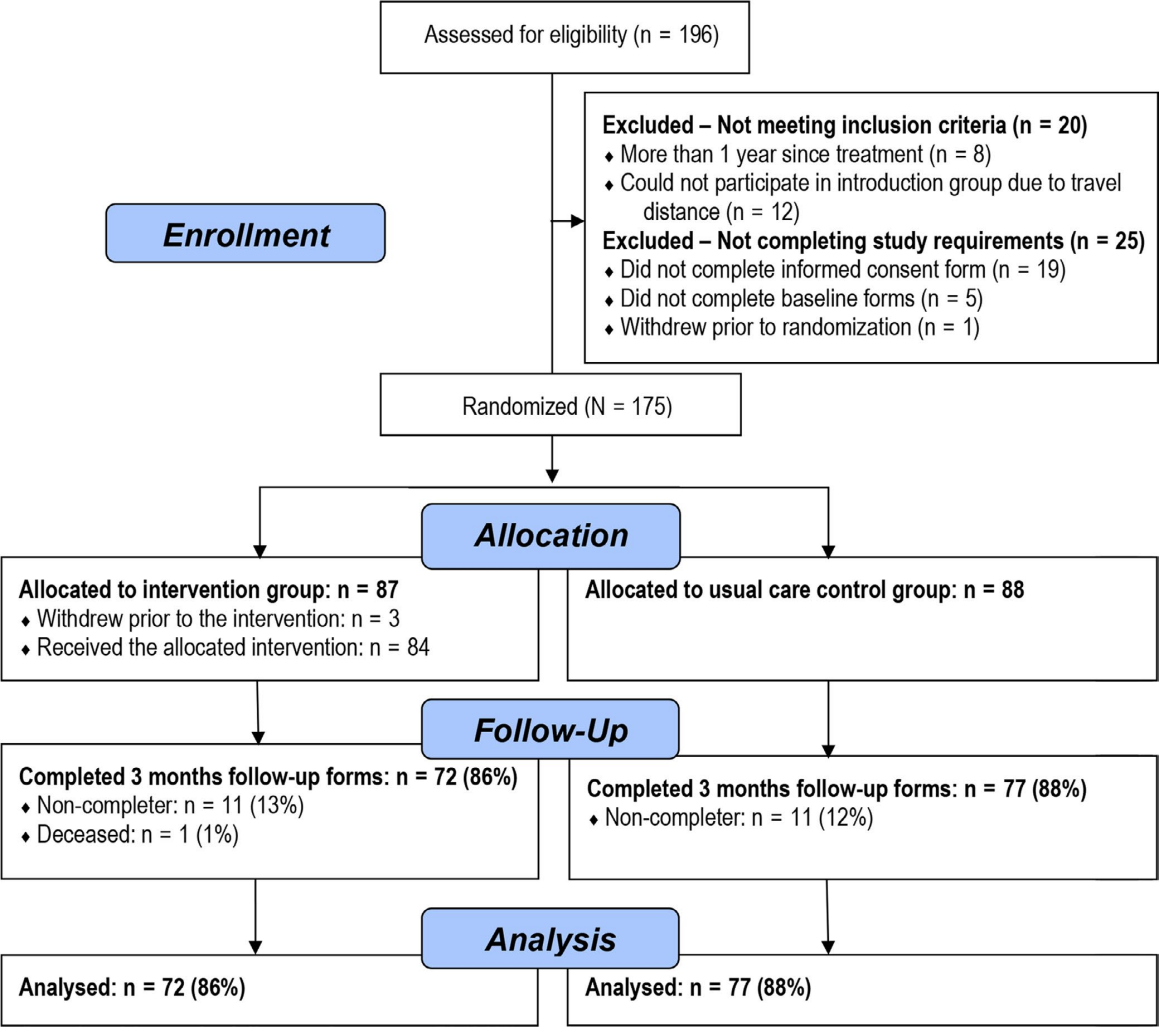
Participant trial flow

The trial flow chart (see Figure 2) shows recruitment and retention from baseline to 3‐month follow‐up. In total, 175 participants with a range of cancer diagnoses were enrolled in the study. The primary reason for ineligibility was travel distance to the single face‐to‐face introduction session. Three participants allocated to the interventions group were unable to attend the face‐to‐face introduction session due to disease progression and were therefore excluded from the study, leaving a total study sample of 172 participants. The majority of the study sample were recruited by medical center staff (102/172; 59%), and the remainder of the sample through social media (70/172; 41%). Employment status was the only significant difference in demographic attribute/user statistics observed between the two types of recruitment (ie, clinical staff vs social media recruitment). In the group recruited through social medial, more people reported engaging in full‐time/part‐time work (22/36; 61%), fewer were on sick leave/disability benefits (45/120; 38%), and fewer reported being retired/other (3/16; 19%), compared to the group recruited by clinic staff (*P *= .007).

Participants (N = 172) mean age at enrollment was 52 (SD 11.3) years. Breast cancer was the most common cancer type (48%), followed by brain (8%), prostate (6%), and lymphoma (5%). At time of enrollment, the majority of participants were married or cohabitating (70%), female (82%), reported a university/college education (81%), and using sick leave/disability benefits (70%) (see Table 1). No significant differences were revealed between the study arms within sociodemographic‐ and disease‐related measures.

### 3.2 System use, usefulness, and ease of use

Of the 84 participants in the intervention group, 49 completed at least seven of the 10 modules within the 3‐month study period, yielding a 58% completion rate. The top three repeated exercises during the 3 months were as follows: diaphragmatic breathing (166 repetitions), progressive muscle relaxation (152 repetitions), and a breathing exercise to reduce stress (152 repetitions). Of the 72 intervention group participants completing the 3‐month ratings, 81% viewed the intervention as useful (ie, “totally agree” or “agree”; 58/72), 94% viewed it as easy to use (68/72), and 97% agreed the *StressProffen* intervention contained easily understandable exercises (70/72).

### 3.3 Group differences

When assessing the change from baseline to 3 months, and according to intention‐to‐treat analysis, the *StressProffen* intervention arm reported significant reductions in perceived stress (mean differences [MD] = −2.8, 95% CI [−5.2 to −0.4], *P *= .022) 3‐months post‐intervention compared to the usual care control group (see Table 2). There was no statistically significant change in the level of anxiety (*P *= .197) or depression (*P *= .119). Intervention effects on HRQoL varied by subscale. Statistically significant differences were observed for four of eight HRQoL domains (Role Physical MD = 17.7, 95% CI [3.7‐31.3], *P = .*013; Social Functioning MD = 8.5, 95% CI [0.7‐16.2], *P = *.034; Role Emotional MD = 19.5, 95% CI [3.7‐35.2], *P = *.016; Mental Health MD = 6.7, 95% CI [1.7‐11.6], *P = *.009) with the intervention group reporting better status than the control group. Participants in the *StressProffen* arm reported improved scores relative to the control arm on all other measures, although not significant.

**TABLE 2:** Primary and secondary outcomes

	Intervention (n = 72)	Control (n = 77)	Between‐group differences	Effect size β	*P* value
	Mean (95% CI)	Mean (95% CI)	MD (95% CI)		
Perceived stress (PSS‐14)					
Baseline	27.5 (25.5‐29.4)	25.8 (23.9‐27.7)			
3 months	22.5 (20.7‐24.3)	23.6 (21.6‐25.6)			
Change from baseline	−5.0 (−7.0 to −3.0)	−2.2 (−3.6 to −0.8)	−2.8 (−5.2 to −0.4)	−.188	.022
Anxiety (HADS‐A)					
Baseline	9.0 (8.0‐10.0)	8.8 (7.8‐9.7)			
3 months	6.7 (5.8‐7.7)	7.3 (6.3‐8.2)			
Change from baseline	−2.3 (−3.1 to −1.4)	−1.5 (−2.3 to −0.8)	−0.7 (−1.9 to 0.4)	−.106	.197
Depression (HADS‐D)					
Baseline	5.7 (4.9‐6.5)	5.2 (4.3‐6.1)			
3 months	4.5 (3.7‐5.2)	4.7 (4.0‐5.5)			
Change from baseline	−1.2 (−1.9 to −0.5)	−0.5 (−1.1 to 0.2)	−0.8 (−1.7 to 0.2)	−.128	.119
HRQoL (RAND‐36)					
Physical functioning					
Baseline	72.0 (67.3‐76.8)	80.3 (75.6‐85.0)			
3 months	74.7 (69.7‐79.7)	76.7 (71.4‐82.0)			
Change from baseline	2.6 (−2.1 to 7.4)	−3.6 (−8.5 to 1.2)	6.3 (−0.5 to 13.0)	.150	.068
Role physical					
Baseline	19.8 (12.2‐27.4)	40.6 (31.1‐50.0)			
3 months	29.2 (20.2‐38.1)	32.5 (23.5‐41.4)			
Change from baseline	9.4 (−0.2 to 19.0)	−8.1 (−18.1 to 1.9)	17.7 (3.7 to 31.3)	.203	.013
Bodily pain					
Baseline	58.6 (52.9‐64.3)	64.3 (59.0‐69.6)			
3 months	62.4 (56.4‐68.4)	63.0 (57.4‐68.6)			
Change from baseline	3.8 (−2.5 to 10.0)	−1.3 (−6.1 to 3.5)	5.1 (−2.7 to 12.8)	.106	.197
General health					
Baseline	48.8 (43.7‐53.8)	55.9 (50.7‐61.1)			
3 months	46.4 (41.2‐51.6)	54.6 (49.6‐59.7)			
Change from baseline	−2.4 (−6.1 to 1.4)	−1.3 (−5.0 to 2.4)	−1.1 (−6.3 to 4.2)	−.033	.688
Vitality					
Baseline	37.3 (32.5‐42.0)	46.8 (41.9‐51.6)			
3 months	41.8 (36.7‐46.9)	50.6 (45.6‐55.7)			
Change from baseline	4.5 (0.1‐8.9)	3.9 (0.1‐7.7)	0.6 (−5.1 to 6.4)	.018	.832
Social functioning					
Baseline	50.0 (43.9‐56.1)	63.0 (57.3‐68.7)			
3 months	62.7 (56.3‐69.1)	67.2 (61.9‐72.6)			
Change from baseline	12.7 (6.6‐18.7)	4.2 (−0.8‐9.3)	8.5 (0.7‐16.2)	.174	.034
Role emotional					
Baseline	44.0 (33.8‐54.2)	53.7 (44.0‐63.4)			
3 months	63.9 (54.0‐73.8)	54.1 (44.2‐64.0)			
Change from baseline	19.9 (7.8‐32.0)	0.4 (−9.9 to 10.8)	19.5 (3.7‐35.2)	.198	.016
Mental health					
Baseline	63.7 (59.2‐68.3)	66.8 (63.0‐70.6)			
3 months	73.5 (69.9‐77.1)	69.9 (66.1‐73.7)			
Change from baseline	9.8 (5.9‐13.7)	3.1 (−0.1 to 6.3)	6.7 (1.7‐11.6)	.214	.009

Abbreviations: CI, confidence interval; MD, mean difference; β, standardized coefficient beta.

## 4 DISCUSSION

In the current study, cancer survivors receiving *StressProffen*,^26^, ^27^ an app‐based cognitive‐behavioral stress‐management intervention program, reported significant reductions in perceived stress compared with cancer survivors receiving care as usual only. Results demonstrate that 3‐months access to an app‐based program, delivered in a blended health‐care delivery model (ie, a single face‐to‐face introduction session, two telephone follow‐up calls, and availability of telephonic support assist with technology use) can significantly improve cancer distress. Secondary outcomes demonstrated that four of eight HRQoL domains also improved for cancer survivors in the *StressProffen* arm.

Despite the promising potential of digital solutions to deliver more flexible and easily available interventions, evidence of effectiveness of psychosocial eHealth interventions (eg, web‐ or app‐based) for cancer survivors have been limited and inconclusive.^18^, ^19^, ^22^, ^23^, ^24^ The current study suggests that an app‐based cognitive‐behavioral stress‐management intervention can provide effective, beneficial, and useful support for cancer survivors when delivered within a blended health‐care delivery model.

Effect sizes in the current study are generally small (β = .2 range). However, data variability is large, which may contribute to the smaller effect sizes but also indicate that even though some participants may not have benefited significantly, others likely benefited greatly. Interpretation of changes in scores in terms of clinical significance is not intuitive, relating to the extent of change considered vital or meaningful to patients,^37^ but should not be underestimated for those reporting benefit from *StressProffen*.

Nonsignificant statistical outcomes do not necessarily mean that the intervention was not clinically effective either, as small sample sizes and measurement variability may impact statistical results. As shown in Table 2, although not statistically significant, participants in the intervention group, compared with those in the control group, did report larger decreases in anxiety and depression, and greater improvements in Physical Functioning, Bodily Pain, and Vitality. These tendencies occurred despite the intervention group reporting lower and less improvement in General Health Perception (ie, believing that personal health is poor and likely to get worse) compared with controls. As none of these changes were statistically significant, however, interpretations should be made with great caution.

Face‐to‐face individual or group‐based cancer distress‐management interventions typically involve at least 10 face‐to‐face sessions^10^, ^11^, ^15^ and are not always available. The blended delivery model evidenced in the current study has the potential to increase the reach of efficacious interventions for cancer survivors, being available for use anywhere and anytime, mostly asynchronously and likely with lower health‐care costs.

Intervention adherence is a major contributing factor for interventions to have positive impact.^38^ Unfortunately, adherence and attrition have emerged as significant challenges to the success of eHealth interventions.^20^, ^25^ A systematic review examining web‐based interventions focusing on chronic disease, lifestyle, and mental health illustrated this challenge, showing how approximately 50% of participants in the 83 studies examined adhered to the web‐based interventions in question.^25^ While 58% of RCT *StressProffen* participants completed the program (at least 7 of 10 modules) within the 3‐month study period, the prior pilot study had a higher completion rate (67% completed the program during an 8‐week study period).^27^ The *StressProffen* pilot‐study duration was shorter; however, and participants were involved as stakeholders and encouraged to use the intervention to help the research team refine the *StressProffen* program. In the current RCT, participants were simply informed the team wished to test effects of an app‐based stress‐management program, and not asked to contribute to refinement of the program. The *StressProffen* intervention studies have defined completers as those completing at least 70% of the intervention.^27^ Similar interventions have, however, also used an a priori criterion of completing at least 50% of the intervention to define completers.^39^ If using the 50% completion criterion (ie, completing at least 5 of 10 modules) for the current study, the intervention had a 68% completion rate. There was no correlation between baseline outcome measure levels and number of program modules completed. It should also be noted that despite high attrition being one of the major challenges with eHealth intervention trials in general,^25^ no withdrawals were observed for participants receiving the *StressProffen* intervention in the current study. The question remains, however, whether all cancer survivors may benefit from a psychosocial eHealth intervention such as *StressProffen*. The results from the current study show large variability in between‐group differences, which could indicate that such an intervention has the potential to be of great benefit to some cancer survivors, but likely not all.

### 4.1 Limitations

This study has several limitations. First, recruitment of participants was performed through several channels, including social media. As such, it can be assumed the participants represent a sample from a population of motivated cancer survivors. The current study cannot conclude whether cancer survivors in general would be interested in, or benefit from, such interventions, limiting conclusions about generalizability at this point. Participation in *StressProffen* when recommended as part of cancer care should be examined in future studies. Second, background data on disease and treatment status were based on participants’ self‐report and as such cannot be verified. This was, however, the same for both groups. Third, given that all outcome measures were in the direction of improvement for the intervention group compared with the control group, but not all significant, it is possible that the study could have been underpowered in terms of having a large enough number of participants to detect all potential impact. Future studies could consider testing these hypotheses in a larger sample of cancer survivors. It should, however, be noted that the power in the current study was more than enough to detect significance in perceived stress and several HRQoL factors. Fourth, the large variability in data may have impacted effect sizes, again potentially indicating a number of participants on the small side. However, the large variability in data likely also contributed to the smaller effect sizes, yet indicating that while some participants may not have benefited significantly, others certainly did. Fifth, given the limited number of nonresponders (13.4%) at 3 months, no statistical model could be fit for missing values imputation. However, the nonresponders were equally distributed between groups, and did not differ in terms of age, gender, or marital status. Nonresponders did tend to represent a lower education level, and responders had higher baseline levels of depression (HADS‐D). Nevertheless, simple imputation models are known to increase bias, and imputations were therefore not conducted. Even though a wide variety of cancer diagnoses are represented in the current study, the majority of participants were female and breast cancer survivors. Future studies should strive to include larger and more heterogeneous cancer survivor populations to improve clinical utility and generalizability. Sixth, participants were not blinded to group allocation. This could have affected belief in impact, particularly if participants had already heard about preliminary effects from the pilot study.^27^ Finally, mood was assessed by questionnaires only and a structured clinical interview may have yielded different results in the domains of anxiety and depression, especially in Northern European and other populations where stoicism and lower emotional self‐disclosure may be the norm. Also, the current study did not employ a minimal distress score (eg, at least a score of 3 of 10) as part of the inclusion criteria. Baseline scores were moderate for perceived stress, slightly elevated for anxiety, and low for depression, while baseline scores for HRQoL varied depending on scale. It is possible that a population of cancer survivors with higher baseline distress scores could have benefited even more, impacting the degrees of change from the intervention. The current study examined short‐term (ie, 3 months) effects from an RCT testing the *StressProffen* intervention program. Future analyses will examine maintenance of these effects.

## 5 CONCLUSIONS

App‐based distress‐ and stress‐management interventions such as *StressProffen* have the potential to significantly improve well‐being for cancer survivors, especially when delivered within a blended care delivery model. In the current RCT, participants receiving *StressProffen* reported decreased perceived stress and increased HRQoL after 3 months use. Given face‐to‐face delivery of such interventions is not always available or feasible for cancer survivors; digital interventions such as *StressProffen*, with easy access and use, may provide low cost, highly accessible wide reach support for cancer survivors with unmet psychosocial needs.

## CONFLICT OF INTEREST DISCLOSURE

Dr Clark is a consultant for Roche Diabetes Care GmbH. No other authors made any disclosures.

## AUTHOR CONTRIBUTIONS

EB: Conceptualization, methodology, data collection, supervision, writing (original draft, review, and editing), and final approval for manuscript submission. SLE, MMC, MAA: Conceptualization, methodology, writing (review and editing), and final approval for manuscript submission. CV, MC: Methodology, writing (review and editing), and final approval for manuscript submission. LSN: Conceptualization, methodology, supervision, writing (original draft, review, and editing), and final approval for manuscript submission.

## Data Availability

The data that support the findings are not publicly available due to privacy or ethical restrictions.
